# Ondine’s curse, a fatal infarction diagnosed by polysomnography and saved by ventilation: a case report

**DOI:** 10.1186/s41983-021-00326-z

**Published:** 2021-06-12

**Authors:** Hany Aref, Tamer Roushdy, Amr Zaki, Nevine El Nahas

**Affiliations:** Neurology Department, Faculty of Medicine, Ain Shams University, Cairo, Egypt

**Keywords:** Ondine’s curse, Lateral medullary infarct, Posterior circulation stroke, Sleep apnea

## Abstract

**Background:**

Lateral medullary syndrome causing Ondine’s curse is a rare yet fatal brainstem infarction. Any patient presenting with lateral medulla infarction ought to be well observed and a polysomnography must be ordered for him.

**Case presentation:**

A patient presenting with Ondine’s curse is dealt with through polysomnography as a diagnostic procedure that was followed by tracheostomy with portable ventilator and cardiac pacemaker as a therapeutic maneuver which ultimately preserved his life.

**Conclusion:**

Lateral medullary syndrome infarct could be a life-threatening stroke if not diagnosed and managed properly.

## Background

The medulla oblongata controls vasomotor and respiratory functions. It is considered the primary respiratory control center, as it sends signals from the respiratory central pattern generators to muscles controlling breathing [1].

Stroke involving the lateral medulla oblongata is named Wallenberg syndrome (lateral medullary syndrome) [2].

Lateral medullary syndrome (LMS) typically presents with hiccup, vertigo, nystagmus, vomiting, dysphagia, dysarthria, and dysphonia that usually resolves without fatal sequel. Two to 6% of LMS presents with disturbance in vital functions causing central apnea, bradycardia, and hypoventilation (Ondine’s curse) that is fatal if not properly identified and managed [3].

Ondine’s curse is named after a mythical story of a man who was doomed to a life where he keeps breathing only while awake and conscious. So there was always a choice between sleeping and remaining alive. In the current case report, we present a case of Ondine’s curse due to LMS.

## Case presentation

The case includes a 57-year-old male, diabetic, hypertensive, with recurrent cerebrovascular strokes dating 2013 and 2016 (modified Rankin score (mRS) 1).

In August 2018, he presented to the emergency room (ER) with sudden onset, 11 h duration of swaying of gait to the left, vertigo, nausea, and vomiting followed by dysphagia to fluids with nasal regurgitation, hoarseness of voice, and partial ptosis of left eye secondary to partial involvement of descending sympathetic tract (National Institute of Health Stroke Scale (NIHSS) 5).

**Fig. 1:**
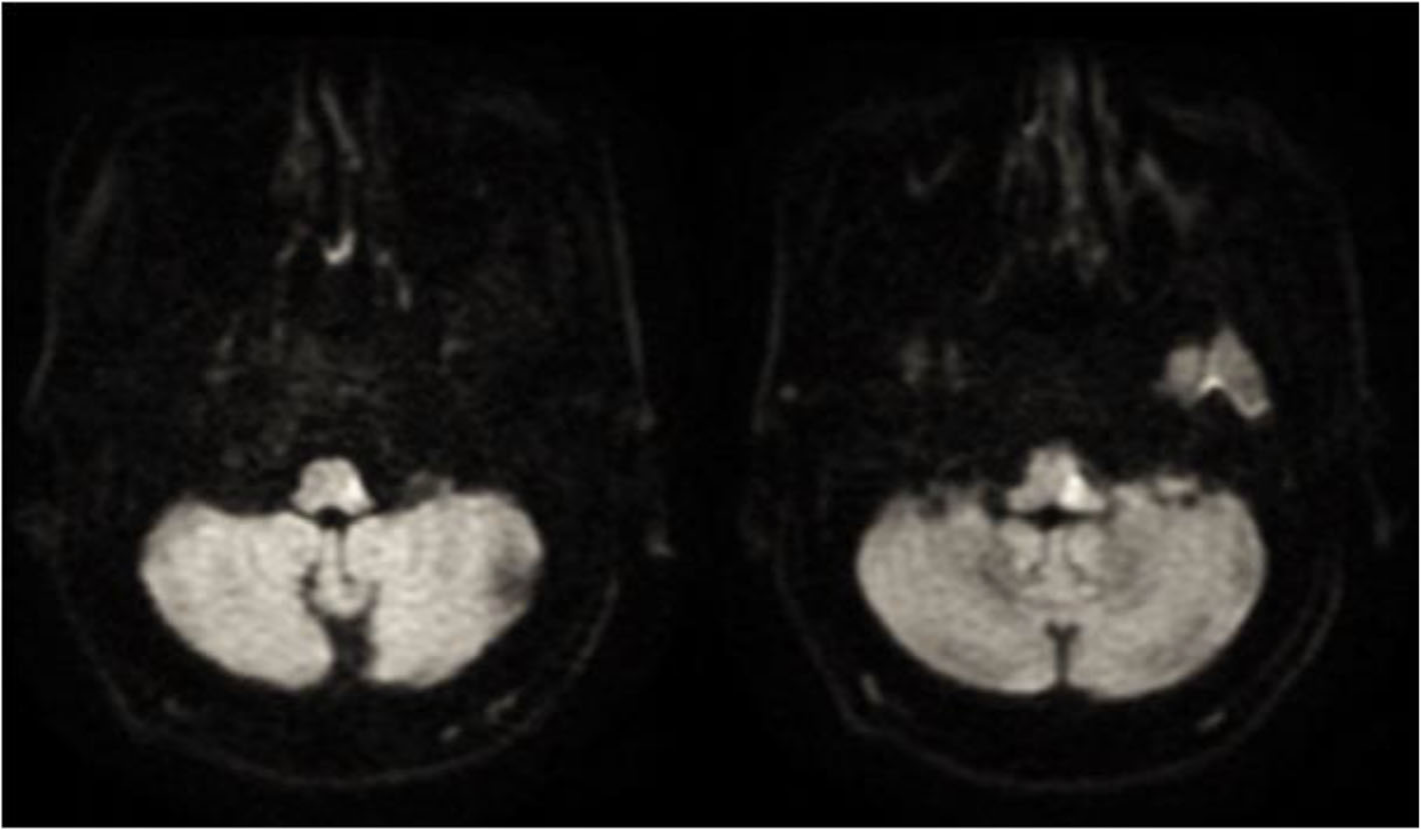
Left lateral medullary infarction along axial magnetic resonance imaging

MRI brain with diffusion-weighted imaging (DWI) revealed left lateral medullary infarction (Fig. 1).

Few hours after admission, he suffered from tachypnea with drop of oxygen saturation to 60%, which was followed by bradycardia (17 beats per minute) and gasping. The patient was rushed to the intensive care unit (ICU) and intubated for assisted ventilation using bi-level positive airway pressure mode (BiPAP) for 24 h after which he became stable and extubated.

Twelve hours later, the patient developed cardiopulmonary arrest and was subjected to cardiopulmonary resuscitation and assisted ventilation (BiPAP) mode. Two days later, he was shifted to continuous positive airway pressure (CPAP); however, he suffered recurrent attacks of apnea at night and was placed on BiPAP mode.

Electrocardiogram (ECG) and cardiac enzymes as well as coagulation profile and D-dimer were within acceptable limits, carotid duplex revealed diffuse atherosclerosis, ECHO showed grade I diastolic dysfunction and mild concentric left ventricular hypertrophy, glycated hemoglobin was 8.5, and lipid profile was normal. Follow-up MRI was stationary and chest X-ray was free.

A week later, he was extubated and was stable on room air. Polysomnography was done prior to discharge and showed an increased percentage of rapid eye movement (REM) sleep. However, after 90 min from sleep initiation, he developed prolonged central apnea (102 s) with a drop of oxygen to 49% and bradycardia with average heart rate of 39.3 beats per minute, all of which were corrected by re-administration of assisted ventilation (Table 1).

**Table 1:** Polysomnography; central apnea report

Central Apnea summary	Total	With HR drop	With SAT drop
Total number	27	10	24
Max length (s)	102.5	102.5	102.5
Apneas preceded by sigh	1	0	0

*Max* maximum, *HR* heart rate, *SAT* saturation

After consultation, tracheostomy with valve to aid in speech and a permanent cardiac pacemaker to manage bradycardia attacks were applied. The patient was discharged on portable ventilator with BiPAP mode (Philips trilogy 100, USA) during sleep and was stable over 2-year follow-up.

## Discussion

Stroke remains a major cause of mortality and morbidity worldwide. Posterior circulation strokes (PCS) account for 20% of all strokes. LMS represents only 2–3% of ischemic strokes [4, 5].

Respiratory complications including hypoventilation syndrome (Ondine’s curse) rarely occur with LMS yet it is fatal if not detected and managed properly [6].

In this case report, we presented a male patient, with prolonged uncontrolled diabetes (glycated hemoglobin 8.5) and hypertension. In previous reports male gender, poorly controlled diabetes and history of hypertension were risk factors contributing to the development of sleep-disordered breathing including Ondine’s curse [7]. Chronic diabetes and hypertension increase the peripheral burden on the respiratory system causing damage to pulmonary capillaries (microangiopathy), myopathy, and autonomic neuropathy. When central hypoventilation sets in, it can cause further respiratory decompensation [8].

Thus, patients with LMS who are at high risk ought to perform polysomnography, even if they showed no breathing problems while awake, so as to detect central apnea and reach a proper management plan. Assisted ventilation with CPAP might be mandatory as a step prior to tracheostomy with usage of portable ventilator thereafter especially during sleep time [9, 10].

In our case, polysomnography revealed the presence of severe sleep apnea with a low baseline oxygen saturation (88%) and frequent dips reaching 49% which suggested a sleep hypoventilation syndrome.

The percentage of REM sleep was high, which was attributable to irregular sleep with insufficient REM periods during the preceding days leading to this REM rebound.

The respiratory events were of the central type with a lack of thoracoabdominal efforts.

## Conclusion

To our knowledge, this is the first case of Ondine’s curse to be reported in Egypt with a full description as regards course and investigations that led to a proper management plan.

## Data Availability

The corresponding author takes full responsibility for the data, has full access to all of the data, and has the right to publish any and all data separate and apart from any sponsor.

## Abbreviations

LMS: Lateral medullary syndrome
mRS: Modified Rankin score
ER: Emergency room
NIHSS: National Institute of Health Stroke Scale
DWI: Diffusion-weighted imaging
ICU: Intensive care unit
BiPAP: Bi-level positive airway pressure
CPAP: Continuous positive airway pressure
ECG: Electrocardiogram
REM: Rapid eye movement
PCS: Posterior circulation stroke
